# Destruction of the craniofacial skeleton in the child caused by an orbital tumor

**DOI:** 10.1007/s00381-014-2540-2

**Published:** 2014-09-27

**Authors:** Grzegorz Goncerz, Janusz Skrzat, Małgorzata Kołodziej, Jerzy Walocha

**Affiliations:** 1 Department of Anatomy, Collegium Medicum, Jagiellonian University, Kopernika 12, 31-034 Kraków, Poland; 2 Department of Anthropology, Jagiellonian University, Gronostajowa 9, 30-387 Kraków, Poland

**Keywords:** Retinoblastoma, Rhabdomyosarcoma, Neuroblastic tumor, Intraocular malignancy, Skull destruction

## Abstract

**Purpose:**

The aims of this paper are to describe the morphological alterations within an infant craniofacial skeleton caused by an orbital tumor and present how the bone reacts in contact with a spreading tumor mass.

**Methods:**

A study was performed on the dry skull of a child at the age of approximately 2 years. Morphological alterations of the craniofacial skeleton were analysed by visual inspection, and the intracranial cavity was examined with the aid of a digital camera. Subsequently, the skull was examined using computed tomography.

**Results:**

The skull was identified as having unilateral symptoms of orbital destruction caused by a malignant tumor, probably retinoblastoma or rhabdomyosarcoma. The left orbit and surrounding bones showed extensive malformation caused by the invading tumor. Profound deformities were also observed in the nasal cavity, which was partially occluded by the collapsed medial wall of the left orbit. The tumor extended to the wall of the orbit, spread out of the orbit, penetrated to the anterior cranial fossa, and probably invaded the brain.

**Conclusions:**

Extensive pathological cranial destruction and possible metastases to inner organs suggest that the orbital tumor was the cause of death. Anatomical alterations observed in the craniofacial skeleton indicate a highly aggressive character of the orbital tumor.

## Introduction

Typical ocular neoplasms which originally develop in the eye cells include retinoblastoma and melanoma. These are non-metastasizing neoplasms, since the eyeball is surrounded with membranes which are difficult to penetrate; also, the inside of the eye does not contain its own lymphatic system, which would favor the spread of neoplastic cells to other organs [6].

Rich blood supply and relatively slow blood flow in ocular blood vessels contribute to the formation of metastatic foci in the eye. Hence, most frequently diagnosed ocular and orbital neoplasms are in fact metastatic forms of tumors in soft parts [12, 32]. Neoplasms occur relatively rarely within orbital sockets. The highest incidence is reported in early childhood, with a noticeable decline in the number of cases in later periods. The disease process of cancer in children is somewhat different to that of adults [24]. Most lytic lesions within the orbit in young children is benign and does not pose a threat to the eye and does not affect the process of seeing [24, 31]

Tumors diagnosed within orbital sockets can be divided into three groups. The first group includes the so-called primary tumors related to neoplastic lesions in the cornea, conjunctiva, and intraocular tumors. Another group comprises tumors which are metastatic forms from adjacent anatomical structures. Such forms include metastatic tumors from the nose, paranasal sinuses, nasopharynx, or neoplasms of the skin surrounding the orbit. Finally, the orbit or its surrounding area may contain lesions of various intensity resulting from malignant metastases from soft parts [8, 29]. The types of neoplasms which occur in children slightly vary in terms of histological type and image from the ones which afflict adults. In children, the most frequent type of tumors are primary tumors, i.e., located in the orbit itself or in the close vicinity of the orbit and connected with the structures of the organ of sight. About 10–30 % of all orbital neoplasms are malignant tumors [6].

One of the most frequent tumors in early childhood, afflicting boys more often than girls, is rhabdomyosarcoma, a benign neoplasm of soft tissues originating from skeletal striated muscles [7, 33]. It makes for 5 % of all tumors forming in the orbital vicinity. The tumor most frequently occurs in the cardiac muscle. Apart from the heart, it may also appear in the head and the neck, and in small children, in the orbital area. In over a third of all cases, rhabdomyosarcoma is located in the head and neck areas, as well as the orbital area; these locations are the primary foci of the neoplasm. The neoplasm may also spread secondarily to the orbit from the nasopharynx, pterygopalatine fossa, infratemporal fossa, or paranasal sinuses (the so-called parameningeal sites) or as a site of metastasis.

Other neoplastic lesions observed in children include vascular tumors located in the vicinity of the orbit and the eye. Congenital neoplasms, e.g., rhabdomyosarcoma activated in the same age group, include venous-lymphatic malformations and lymphangiomas [6]. Another similar neoplasm located originally in the orbit is the retinoblastoma.

Retinoblastoma is the intraocular malignant tumor occurring mostly during infancy and childhood. It arises from neuroectodermal cells that are destined to become photoreceptors of the retina [22]. The annual incidence of retinoblastoma is l/15,000–1/30,000 live births [3, 30, 35]. The disease usually afflicts children before the age of 5 years [3, 38], but adult cases have also been clinically recorded [20] Families that carry the RB gene mutation are particularly prone to retinoblastoma [10, 22]. The tumor occurs more often unilaterally (2/3 to 3/4 of cases) than bilaterally [1, 3]. Bilateral and multifocal unilateral tumors are hereditary. According to Tamboli et al. [35], there are no differences in incidence of retinoblastoma by sex, race, and preference for either the right or left eye. Clinical symptoms can be: headache, nausea, unsteady gait, visual field defects, hypopyon, hyphema, buphthalmia [3, 5, 25]

The aim of this paper was to present the morphological alterations within craniofacial skeleton of the child caused by the orbital tumor. Hence, we tried to find out which ocular neoplasm could be a possible reason of the observed defects.

## Material and method

A skull afflicted with an ocular tumor belongs to the cranial collection, which is housed in the Department of Anatomy of the Medical College of the Jagiellonian University. The skull is well preserved and shows no traits of any cranial deformities, except those caused by the tumor. Remnants of the soft tissues were observed within the orbital cavities. They are dried and locally delaminated from the bone. Visual inspection of the skull revealed its child character but the sex is unknown. The following features refer to the early age of individual: the frontal fontanelle is well developed, while the occipital, sphenoid, and mastoid are obliterated. The lower portion of the metopic suture is fused. The other sutures of the cranial vault are opened. There are prominent parietal tubers, bones are thin and delicate, neurocranium is much bigger than viscerocranium. Both upper incisors (I^1^, I^2^) and molars (M^1^) came through the maxilla, but the upper canines were already teething. The mandible is not preserved therefore its dentition was not assessed.

Taking into account the overall cranial morphology, stage of teeth eruption, state of the sutures of the vault, and presence of the anterior fontanelle, we presume that the child’s age at death was approximately 21–24 months [4].

The interior of the cranial cavity was investigated by a small digital camera which was introduced into the skull through the foramen magnum. Particular attention was paid on the posterior wall of the orbit afflicted with a tumor. Digital images obtained from the camera were processed with ImageJ program which is a public domain, Java-based image processing software (US National Institutes of Health; http://rsb.info.nih.gov/ij). The size of the pathologic change within the facial skeleton was expressed by the biggest horizontal and vertical diameter, measured by the digital sliding caliper.

In an attempt to study the inner structure of the craniofacial skeleton, computed tomography was employed using a ten-row CT scanner (Somatom Sensation 10, Siemens). The parameters of the CT study were as follows: matrix 512; exposure factors 120 kVp, 120 mAs; slice thickness 0.6 mm.

The computed tomography yielded images in a very wide range of greyscale. Therefore, the brightness and contrast of the slices viewed was adjusted to display the details of the osseous structure. A linear intensity window setting technique was used to view CT scans in order to provide adequate contrast and ensure a good quality of the visualized osseous structures.

Computed tomography of the skull was also employed to analyze the inner structure of the calvarial bones and to observe potential changes of the diploe and tables of compact bone caused by aging processes. In order to perceive more details in the images, we applied a zoom factor of 1.5–2.0. This facilitated a detailed analysis of the relationship between the diploe and the outer and inner table of the compact bone.

## Results

**Fig. 1:**
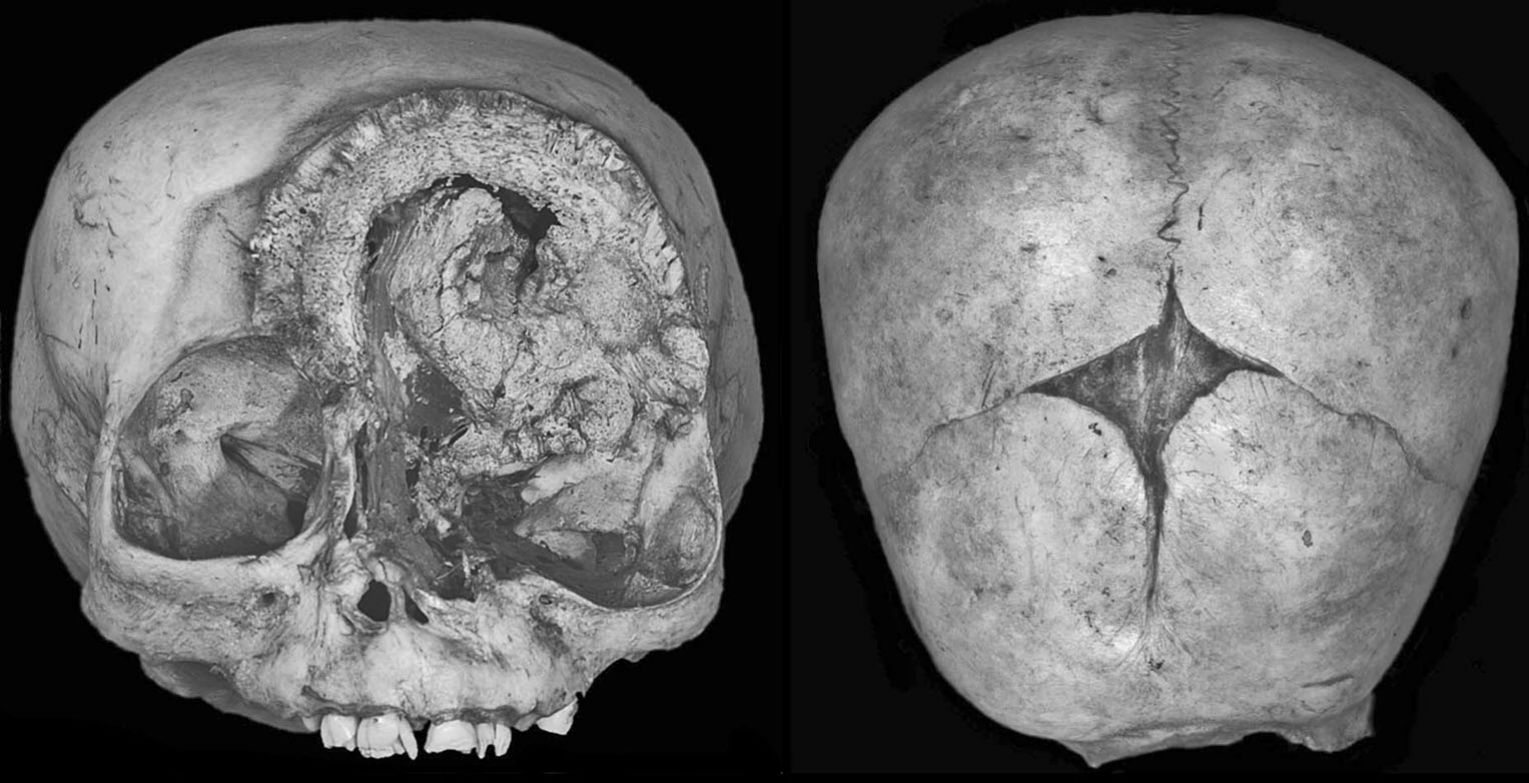
Anterior and superior view of the skull afflicted with tumor’s changes

Pathologic changes leading to abnormal anatomy of the orbit caused by the tumor are observed both in superior and anterior aspect of the skull. The most profound deformities caused by the tumor activity occurred within facial region. Morphological changes refer not only to the malformation of the orbital cavity but mostly to the left part of the face, which was afflicted with the disease. The size of the pathologic change within the facial skeleton was measured as 600 × 480 mm. It comprises mainly the left orbit, left maxilla, left zygomatic bone, and also the orbital part of the frontal bone. The right orbit is almost not altered (Fig. 1).

The apparent superior margin of the left orbit is turned inside out, widened, and protruded so the vault in the region of the forehead is compressed. The frontal bone along this margin is markedly darker in color than normal bone. Destroyed anterior edge of the frontal squama shows extensive hypertrophy of the diploe that was visible because of the destruction of the external lamina of the compact bone. The diploe of the left orbital wall was strongly compressed and locally shows perpendicular lamelation.

**Fig. 2:**
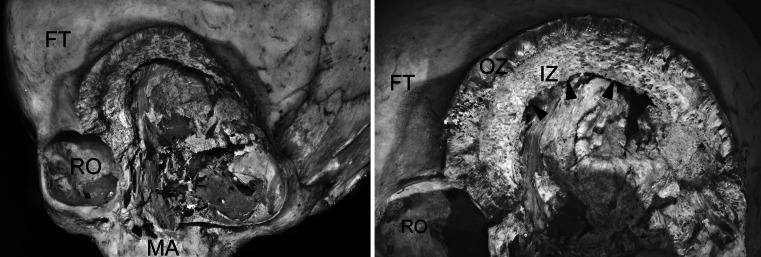
Closeup view on the left orbit eroded by the tumor. Note malformed supraorbital edge showing distinct organization of the osseous structure (*FT* frontal bone, *MA* maxilla, *RO* right orbit, *OZ* outer zone of vertical trabecula, *IZ* inner zone of porous bone), the *arrowheads* indicate cleft of the crescents shape penetrating to the cranial cavity

**Fig. 3:**
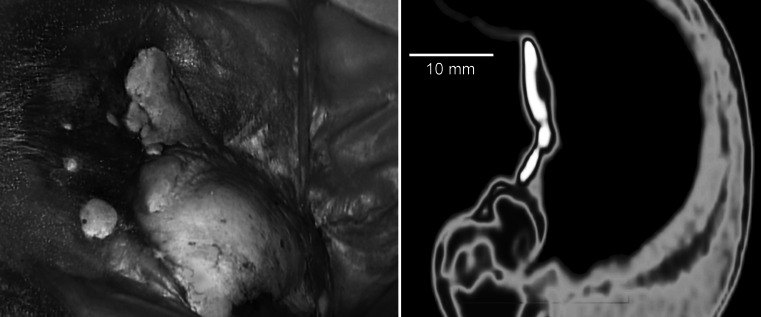
Intracranial view of the calcified spots of the dura mater adjacent to posterior wall of the orbit afflicted with a tumor. CT scan presents calcification of the dura mater

In the anterior aspect, the frontal bone forming supraorbital edge and the roof of the orbit is strongly remodeled. It contains two distinct zones of the bony tissue (Fig. 2). The inner zone is less dense and porous while the outer zone appears denser, probably because of vertical condensation of the bony trabeculas. Underneath there is an atypical cleft of the crescents shape, which penetrates to the anterior cranial fossa so the dura mater of the brain is visible. This cleft extends from the midplane towards the left frontal tuber (Fig. 3).

The supero-lateral wall of the orbit is extensively eroded that manifests in well-visible structure of spongy bone. Posteriorly, the lateral orbital wall is malformed and exists in the form of the thin ossified membrane. It continues towards the optic canal, which is occluded. Numerous and irregular bony granulations extend from the postero-lateral wall of the left orbital cavity. They partially covered up the left superior orbital fissure, forming a narrow protuberance. These structures are of unclear origin and might have resulted from ectopic ossification of the soft tissue that covers the orbital wall or invasion of tumor. The inferior edge of the left orbit is extremely thin and bulged downward and forward, thus lowering the position of the left infraorbital foramen.

The medial wall of the orbit is extremely thin with single perforations that communicate to the nasal cavity, particularly in the lower and posterior part. Hence, the orbital plate of the ethmoid bone is deepened towards the midplane.

The nasal cavity is strongly deformed and partially occluded by the invaginated medial wall of the left orbit towards nasal septum. The left lateral margin of the piriform foramen is dislocated so it adheres to the nasal septum. This condition caused a significant reduction of the capacity of the nasal cavity and considerable malformation of the piriform foramen.

Visual inspection of the intracranial cavity performed by the digital camera revealed presence of completely ossified spots within the dura mater. This observation was also confirmed by the CT examination.

CT imaging revealed regular hyperostosis of the inner surface of the frontal squama with bleared compact tissue and a visible difference between the structure of the hyperostotic tissue and the diploe. In addition, irregular hyperostosis and calcifications in the orbital apex without narrowing of the optic canal as well as small calcifications adjacent to detached dura mater at the level of the orbital apex. The CT image also shows irregular rim of calcification around osteolytic destruction of the superior margin of the orbit with a blearing of compact tissue.

Presented skull afflicted with tumor changes reveals how aggressive the neuroblastic tumor can be and what is the morphological response of the bones when subjected to its activity. Our diagnosis inclines that craniofacial destruction in the analyzed skull might be a possible effect of the retinoblastoma or rhabdomyosarcoma.

## Discussion

The detailed analysis of lesions by means of computed tomography did not allow us to provide a clear diagnosis of the case discussed in this study. Notwithstanding the above, in their diagnosis, the authors are inclined towards advanced lesions characteristic of rhabdomyosarcoma. Still, a different neoplasm, i.e., retinoblastoma, which also typically occurs in the first decade of human life, cannot be excluded. An individual affected by this condition frequently shows such symptoms as leukocoria and strabismus [2]. Patients with advanced retinoblastoma present with orbital mass, blindness, and proptosis. They frequently have the following signs and symptoms of central nervous system disease: seizures, irritability, or coma [1].

The clinical classification of retinoblastoma comprises four stages: stage I, intraocular disease; stage II, orbital extension; stage III, central nervous system metastasis; and stage IV, hematogenous metastasis [11].

According to Tateishi and colleagues [37], retinoblastoma is divided into three types: type A, intraocular tumor; type B, intraorbital tumor with spread into the optic nerve; and type C, tumor extending to lateral aspect the orbit and invading the brain via the sphenoid bone.

The skull, if regarded as a case of retinoblastoma, may potentially be classified as type C, a tumor extending to the orbital wall and probably invading the brain.

Extensive pathological alterations of the skull morphology and possible metastasis to inner organs allow concluding that retinoblastoma was the reason of death of the investigated child. Almost all untreated patients with retinoblastoma die of intracranial dissemination of the disease within 2 years [19]. It appears that this period of potential survival corresponds to the estimated age at death in our case.

Hence, premature metopic craniosynostosis and frontal sinus aplasia can be observed [38]. As the retinoblastoma develops inside the eye, it progressively destroys all surrounding structures and spreads also over the bones of the facial skeleton. In the studied skull, the area of the frontal bone, which border with edges of the pathologic change, have darker color than normal bone. It might be caused by infiltration and bone atrophy because of compression by the tumor mass.

Perpendicular lamelation of the spongy bone within left supraorbital region resembles altered pattern of the diploe that can be found in people afflicted with anemia. In this disease, red marrow hyperplasia causes widening of the diploic space, and the outer table of compact bone thins or is completely obliterated. Hence, thickening of the trabeculae and their arrangement in parallel rows within the diploe may occur [13, 21, 27] and describe the changes in the diploe associated with anemia like the *hair-on-end* sign (thin vertical striations).

In our case, possible extensive function of the red bone marrow related to the tumor activity could cause an overgrowth of the diploe and alteration of its structure. Some authors have enhanced activity of bone marrow cancer, combined with the other, which is rhabdomyosarcoma. In histological researches, features of infiltrated marrow were so characteristic that the diagnosis of alveolar rhabdomyosarcoma could be made [26]. Hence, in our case, the observed changes in hypertrophic dipoles can be an evidence for rhabdomyosarcoma. This might have been also a result of the tumor expansion and compression on the bone leading to internal remodeling. The marrow hyperactivity of the cranial bones can be a response to local invasion of the tumor.

Retinoblastoma can invade surrounding bones by infiltration or metastasis. In rare cases, retinoblastoma gives metastases to the maxilla and mandible. Bone destruction can also concern the upper molar region around the first permanent molar, and it can spread into the maxillary sinus eroding its wall [34]. The investigated skull does not show any noticeable traces of metastasis within alveolar processes of the maxilla. Although the shape of the orbital part of the maxilla was altered, the bone structure seems to be intact by the tumor. Similarly, the teeth of the maxilla present apparently normal external morphology. Considering the above arguments in favor of metastases and lesions within the maxilla, the question on whether the analyzed case may be classified as retinoblastoma is disputable. Please note that the lack of extracranial bones, which could be useful in verifying potential hematogenous metastases to remote locations [11].

A malignant soft tissue tumor which originates in muscles and is the most frequent neoplasm among young children is more likely. Rhabdomyosarcoma accounts for 50 % all soft tissue sarcomas and about 10 % of all malignant extracranial solid tumors. Rhabdomyosarcoma is more frequent in males than in females; in this case, unfortunately, the sex is unknown. Lack of an extant mandible or complete deciduous dentition made it impossible to establish the sex of the subject by morphometric or macroscopic means based on mandibular morphology. Due to an unverified origin of the skull and high probability of sample contamination, no attempt was made to establish the sex by genetic means.

Rhabdomyosarcoma may occur in various locations but most frequently the tumor develops in the orbit, and the affected individual shows such symptoms as the ptosis of the upper eyelid with simultaneous narrowing of the palpebral fissure, exophthalmos, and visual impairments due to the pressure exerted on the heads of the optic nerve [39]. The close proximity of the tumor may cause a decrease or loss of pressure in the nerve, resulting in papilloedema. The discussed case revealed irregular hyperostosis and calcifications in the orbital apex without narrowing of the optic canal.

No calcifications are usually obtained in the tumor; if they occur, they are related to the destruction of the adjacent bone [16]. For this subject, CT imaging allowed us to identify calcifications not only in the optic canal but also small calcifications adjacent to detached dura mater at the level of the orbital apex.

Tumor etiology is not completely known, with genetic factors assumed to play a significant role in the development of some its forms. The embryonic form develops usually in the orbit. An expanding tumor forms a large, compact, and irregular mass of soft tissue and affects the adjacent bone tissues. In the present study, the destruction of the subject’s orbital walls was and adjacent bones extensive. As reported by Sohaib et al. [28] and Shields et al. [29], most cases are located in the superonasal part of the orbital quadrant. The lesions observed in the infant skull were not limited to the orbit, extending to the nasal cavity, mainly from the left side. The tumor was strongly deformed and partially occluded by the invaginated medial wall of the left orbit towards nasal septum. In more advanced forms, bone erosion may occur; the neoplasm may also infiltrate adjacent paranasal sinuses or the nasopharynx [17, 23]. Jones, together with associate researchers [14], observed rhabdomyosarcoma identified in the orbit and in some cases reported the presence of the tumor in the nose. Conceivably, the tumor in this study developed not only inside the orbit but also metastasized to the nasal cavity. Some authors also concluded that the original form of the tumor may develop in the nasopharynx rather than in the orbit and subsequently metastasize to the orbit [6, 29].

We would like to accentuate that in medical literature, there are not many descriptions of the pathological alterations within the craniofacial skeleton caused by retinoblastoma or rhabdomyosarcoma. Therefore, our case report can be included in the study of the antiquity of tumors. Scant in comparative literature describing lesions in the orbital bones caused by such neoplasms makes it rather difficult to give an accurate and final diagnosis. Any description of those rare cases, in particular rhabdomyosarcoma, focuses on contemporary occurrences diagnosed in the early stages of the disease, when bone destruction is not as extensive [9, 15, 18, 29]. In the work described are changes within the tumor itself and not the changes that are caused by tumor in bone interaction. Changes in the bones were imaged using CT but the resulting image was dark and is not always clear to interpret due to tumor infiltration [36]. Considering the extensive and advanced lesions which affected the child whose skull was analyzed in the present study, it is assumed that the tumor, diagnosed as rhabdomyosarcoma, developed intensively, and was untreated. Consequently, the child died in the first decade of his/her life. This work, containing a detailed description of a single case with advanced neoplastic lesions, may provide new information on the malformation and destruction of the craniofacial region, which can occur as a result of rhabdomyosarcoma.

## References

[1] Abramson DH (1982). Retinoblastoma: diagnosis and management. CA Cancer J Clin.

[2] Abramson DH, Beaverson K, Sangani P, Vora RA, Lee TC, Hochberg HM, Kirszrot J, Ranjithan M (2003). Screening for retinoblastoma: presenting signs as prognosticators of patient and ocular survival. Pediatrics.

[3] Aerts I, Lumbroso-Le Rouic L, Gauthier-Yillars M (2006). Retinoblastoma. Orphanet J Rare Dis.

[4] AlQahtani SJ, Hector MP, Liversidge HM (2010). Brief communication: the London atlas of human tooth development and eruption. Am J Phys Anthropol.

[5] Chantada G, Fandiño A, Manzitti J, Urrutia L, Schvartzman E (1999). Late diagnosis of retinoblastoma in a developing country. Arch Dis Child.

[6] Chung EM, Smirniotoponlos JG, Specht CS, Schroeder JW, Cube R (2007). Pedriatric orbit tumors and tumorlike lesions: nonosesous lesion of the extraocular orbit. Radiographics.

[7] Conneveely MF, Mafee MF (2005). Orbital rhabdomyosarcoma and simulating lesions. Neuroimaging Clin N Am.

[8] Crist WM, Anderson JR, Meza JL (2001). Intergroup rhabdomyosarcoma study – IV results for patients with nonmetastatic disease. J Cin Oncol.

[9] Freling NJM, Merks JHM, Saeed P, Balm AJ, Bras J, Pieters BR, Adam JA, van Rijn RR (2010). Imaging findings in craniofacial childhood rhabdomyosarcoma. Pediatr Radiol.

[10] Friend SH, Bernards R, Rogelj S, Weinberg RA, Rapaport JM, Albert DM, Dryja TP (1986). A human DNA segment with properties of the gene that predisposes to retinoblastoma and osteosarcoma. Nature.

[11] Grabowski EF, Abramson DH (1987). Intraocular and extraocular retinoblastoma. Hematol Oncol Clin North Am.

[12] Gupta A, Das S, Murthy R, Vemuganti GK (2010) Orbital tumors in children. Orbit/Plastic Surgery Sesion – I AIOC 491–493

[13] Hollar MA (2001). The hair-on-end sign. Radiology.

[14] Jones IS, Reese AB, Krout J (1965). Orbital rhabdomyosarcom: an analysis of sixty-two cases. Trans Am Ophthamol Soc.

[15] Karcioglu ZA, Hadjistilianou D, Rozans M, DeFrancesco S (2004). Orbital rhabdomyosarcoma. Cancer Control.

[16] Kodet R, Newton WA, Hamoudi AB, Asmar L, Wharam MD, Maurer HM (1997). Orbital rhabdomyosarcomas and related tumors in childhood: relationship of morphology to prognosis—an intergroup rhabdomyosarcoma study. Med Pediatr Oncol.

[17] Mafee MF, Pai E, Philip B (1998). Rhabdomyosarcoma of the orbit: evaluation with MR imaging and CT. Radiol Clin North Am.

[18] McCarville MB, Spunt SL, Pappo AS (2001). Rhabdomyosarcoma in pediatric patients: the good, the bad, and the unusual. AJR Am J Rentgenol.

[19] Melamud A, Palekar R, Singh A (2006). Retinoblastoma. Am Fam Physician.

[20] Odashiro AN, Pereira PR, de Souza Filho JP, Cruess SR, Burnier MN (2005). Retinoblastoma in an adult: case report and literature review. Can J Ophthalmol.

[21] Olufemi-Williams A, Lagundoye SB, Johnson CL (1975). Lamellation of the diploe in the skulls of patients with sickle cell anaemia. Arch Dis Child.

[22] Pacal M, Brenner R (2006). Insights from animal models on the origins and progression of retinoblastoma. Curr Mol Med.

[23] Panaś M, Wyszyńska-Pawelec G (1999). Rhabdomyosarcoma głowy i szyi w materiale własnym. Czas Stomat.

[24] Penn S, Dunbar MT, (1999) Orbital tumors in children. Clin Eye Vision Care 10:195–204

[25] Provenzale JM, Weber AL, Klintworth GK, McLendon RE (1995). Radiologic-pathologic correlation. Bilateral retinoblastoma with coexistent pinealoblastoma (trilateral retinoblastoma). Am J Neuroradiol.

[26] Reid MM, Saunders PWG, Bown N, Bradford CR, Maung ZT, Craft AW, Malcolm AJ (1992). Alveolar rhabdomyosarcoma infiltrating bone marrow at presentation: the value to diagnosis of bone marrow trephine biopsy specimens. J Clin Pathol.

[27] Sebes JI, Diggs LW (1979). Radiographic changes of the skull in sickle cell anemia. AJR.

[28] Sohaib SA, Moseley I, Wright JE (1998). Orbital rhabdomyosarcoma: the radiological characteristics. Clin Radiol.

[29] Shields CL, Shields JA, Honovar SG, Demirci H (2001). Primary ophthalmic rhabdomyosarcoma in 33 patients. Trans Am Ophthalmal Soc.

[30] Shields JA, Augsburger JJ (1981). Current approaches to the diagnosis and management of retinoblastoma. Surv Ophtalmol.

[31] Shields JA, Bakewell B, Augsburger JJ, Donoso LA, Bernardino V (1986). Space occupying orbital masses in children, a review of 250 consecutive biopsies. Ophthalmology.

[32] Shields JA, Shield CL (2004). Orbital cysts of childhood classification clinical features and management. Surv Ophthalmol.

[33] Shields JA, Shields CL (2003). Rhabdomyosarcoma: review for the ophthalmologist. Surv Ophthalmol.

[34] Taguchi A, Suei Y, Ogawa I, Naito K, Nagasaki T, Lee K, Fujita M, Tanimoto K (2005). Metastatic retinoblastoma of the maxilla and mandible. Dentomaxillofac Radiol.

[35] Tamboli A, Podgor MJ, Horm JW (1990). The incidence of retinoblastoma in the United States: 1974 through 1985. Arch Ophthalmol.

[36] Tang TT, McLeary MS (1999). Imaging of the cranium: pictorial essay. Acad Radiol.

[37] Tateishi U, Hasegawa T, Miyakawa K, Sumi M, Moriyama N (2003). CT and MRI features of recurrent tumors and second primary neoplasms in pediatric patients with retinoblastoma. Am J Roentgenol.

[38] Yue NC, Benson ML (1996). Retinoblastoma. Pediatr Radiol.

[39] Yousem DM, Lexa FJ, Bilaniuk LA, Zimmerman RI (1990). Rhabdomyosarcoma in the head and neck: MR imaging evaluation. Radiology.

